# Strengthening integrated research and capacity development within the Caribbean region

**DOI:** 10.1186/1472-698X-11-S2-S7

**Published:** 2011-11-08

**Authors:** Martin Forde, Karen Morrison, Eric Dewailly, Neela Badrie, Lyndon Robertson

**Affiliations:** 1 St. George's University, Grenada, W.I; 2 University of Guelph, Canada; 3 Universite Laval, Quebec, Canada; 4 The University of the West Indies, Trinidad, W.I; 5 The University of the West Indies, Barbados, W.I

## Abstract

**Background:**

The Caribbean region, like other developing regions of the world, faces significant challenges in conducting research, especially in the context of limited resource capacities and capabilities. Further, due to its diverse and multiple island states, research capacity is scattered and unevenly spread within the region. The Caribbean EcoHealth Programme (CEHP) is a research program that is structured to improve the capacity and capability of health professionals in the Caribbean region to respond in integrative and innovative ways to on-going and emerging environmental health challenges by means of multi-sectoral interventions.

**Methods:**

Core parts of the CEHP’s mission are to (1) conduct collaborative research in areas that the region has identified as critical; (2) build and strengthening integrated approaches to research; and (3) develop and enhance basic research capacity within the Caribbean region.

Fundamental to the success of the CEHP’s human and resource development mission has been its use of the Atlantis Mobile Laboratory (AML). The AML has allowed the CEHP program to move throughout the Caribbean and be able to respond to calls for specific research and capacity building opportunities.

**Results:**

The CEHP’s five main research projects have generated the following results: (1) the Persistent Organic Pollutants (POPs) study has evaluated human exposures to POPs, heavy metals, pesticides, and zoonotic infections; (2) the Burden of Illness (BOI) studies have developed protocols for the testing of foodborne microorganisms, strengthen laboratory analytical capabilities, and determined the prevalence and incidence of food-borne illness; (3) the Rainwater Harvesting (RWH) study has evaluated the microbial and chemical quality of rainwater harvesting systems; (4) the Ecotoxicology Water (ETW) studies have provided much needed data on the quality of recreational and drinking water supplies, and (5) the Food Safety Training Program has developed Diploma and M.Sc Agri-Food Safety and Quality Assurance programmes.

**Conclusions:**

The CEHP program provides a successful example of how a collaborative instead of researcher driven research agenda can lead to not only the generation of needed information, but also leave within the region where the research has been carried out the capacity and capabilities to continue to do so independent of outside interventions.

## Background

Indigenous research is an essential activity that needs to take place within each region of the world if practical and sustainable solutions are to be found. In most low- and middle-income (LMIC) regions such as the Caribbean, there is a growing need to strengthen and operationalize the concept of research that integrates various disciplines, allows for organizational inputs at multiple levels (communities, NGOs, governmental), and incorporates sectoral perspectives in a way that is practical and sustainable [1,2]. Within the Caribbean region, there is also a pressing need to build resource capacity and human capabilities that would allow this region the ability to cope with and handle in a sustainable fashion the numerous challenges it faces [3,4]. The Millennium Development Goals highlight the need to develop a global partnership for development (Goal 8), particularly for small island developing states (Target 8C) [5].

The Caribbean EcoHealth Programme (CEHP) is a 5-year research, resource capacity and human capabilities development program that brings together regional and non-regional academic, governmental, non-governmental, and technical organizations to address key gaps in the Caribbean region as they pertain to human health infections and poisonings [6,7]. Current key gaps in the region’s knowledge base include: exposures to environmental pollutants such as persistent organic pollutants (POPs), heavy metals, and pesticides [8-11]; the chemical and microbial quality of seawater and freshwater sources such as water collected by rainwater harvesting systems [12,13]; and the incidence and prevalence of foodborne burden of illness (BOI) and zoonotic infection diseases in this region [14-18]. The overall mission of the CEHP is to investigate such pressing environmentally-related health problems within the Caribbean region and design effective multi-sectoral interventions. All CEHP program activities are anchored on three core principles: (1) the creation and nurturing of collaborative, multi-disciplinary teams which involve Caribbean and Canadian institutions; (2) the development and enhancement of human capabilities; and (3) the development and enhancement of local resource capacity.

Fundamental to the success of the above–mentioned human and resource developmental goals and objectives has been the use of a three million dollar Atlantis Mobile Laboratory (AML) funded in 2003 by the Canadian Foundation for Innovation and the Quebec government. The AML has been central in allowing the CEHP to meet and fulfill its above stated core principles. It has also allowed the CEHP program to move from one Caribbean island to another and to respond to the call for specific research and capacity building opportunities that encourage professional development within the Caribbean region.

## The CEHP research program and objectives

A fundamental, core objective of the CEHP program is to improve the capacity and capability of public and environmental health professionals in the CARICOM region to respond in integrative and innovative ways to on-going and emerging epidemiological and environmental health challenges by means of multi-disciplinary interventions. In other words, the CEHP’s overarching objective is centered on building within the Caribbean region a multi-disciplinary and sectoral community-of-practice that is capable of pursuing and utilizing integrated approaches in solving public and environmental health problems.

**Figure 1:**
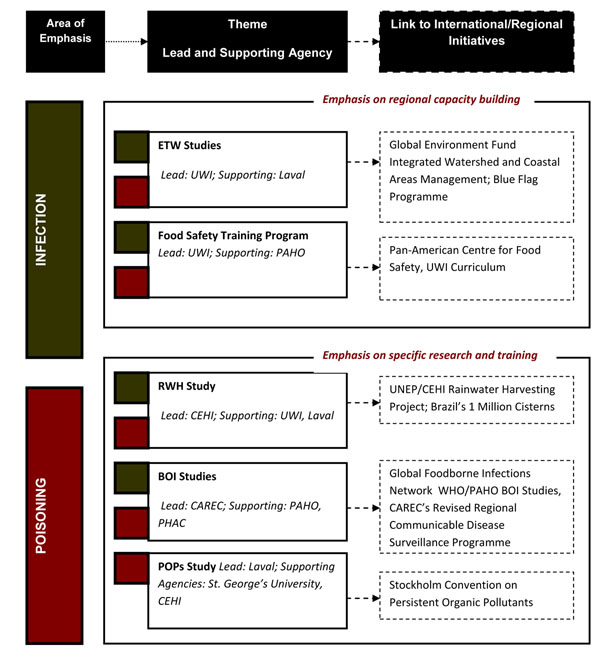
Link between programme themes, partner institutions, and on-going initiatives.

In support of this overarching objective, community(ies) of practice are being formed and strengthened around five main research projects and a number of additional research projects that have evolved over the course of the program. Within each of the CEHP research projects there is thus a local human capability development component. The five main projects are: (1) POPs Study: a study to evaluate human exposures to persistent organic pollutants (POPs), heavy metals such as lead and mercury, pesticides, and zoonotic infections; (2) BOI Studies: studies to evaluate foodborne burden of illnesses (BOI); (3) RWH Study: a study to evaluate the microbial and chemical quality of rainwater harvesting systems; (4) ETW Studies: eco-toxicology water studies to evaluate the quality of recreational and drinking water supplies, and (5) Food Safety Training Program: a distance education degree certification program in food safety and management. As indicated in **Figure**1, each study has a lead organization in charge of overseeing the study and is supported by a range of other organizations. The list of supporting organizations has changed over time as the research programs have expanded and evolved. Further, as highlighted in **Table**1, the CEHP has expanded the number of participating institutions involved in some of the above listed core research programs as well as also initiated and developed other research study programs.

**Table 1:** Emergent research themes, and lead and supporting institutions of the Caribbean EcoHealth Programme.

Emergent Research Themes	Lead (bold) and Supporting Institutions
Ciguatera	**Turks and Caicos MOH**, University of Guelph
Ecotoxicology	**UWI-Cave Hill**, RUSM, Laval University
Indicators of Bathing Water Quality	**UWI-Cave Hill**, RUSM, Laval University
Indigenous Environment and Health	**RUSM**, Kalinga Territory, Trent University
Ethics of International Research	**SGU**, University of Guelph, University of Winnipeg

In addition to the CEHP’s program goal of building and enhancing local and regional human resource capacity and capabilities, a secondary goal is the development of material resource capacity such as laboratory equipment and supplies wherever possible and feasible within the Caribbean region.

### The Atlantis Mobile Laboratory (AML)

The AML is the focal point for a wide range of CEHP activities. The AML is a collection of six twenty-foot containers that have been retrofitted to function as a self-contained laboratory. Three of the containers are outfitted to be chemistry, microbiology, and ecotoxicology laboratories; the other three containers serve as office and living quarters, a service unit which houses the standby generator and workshop tools, and a storage container for bulky items and transshipment of a field vehicle and boat.

The CEHP program has been designed so as to allow the AML to move from one island to another island after staying for a period of approximately eight to ten months. As the AML has moved from one island to another, it has provided an immediate analytical capacity to that island that was not available before. The AML thus provides local governments with a rare window of opportunity to carry out environmental monitoring activities that are demand-driven and guided and based on self-identified national priorities. Most importantly, the AML has allowed the CEHP program to adopt a more collaborative and integrative approach to research instead of research that is researcher driven and typically done in an isolated, non-integrative manner.

The AML has also provided the CEHP program the opportunity to help develop and enhance local laboratory technician capabilities by exposing them to relatively novel analytical techniques. It should be noted that while the AML is the focal point for a wide range of CEHP activities – including research, capacity building, training and promotion and outreach – not all of the CEHP research and capacity building revolves around the lab. The BOI and RWH studies, for example, work directly with national government and regional laboratories and the UWI initiative focuses on its internal lab capacity and curriculum.

## Results and outcomes

### Improving and expanding the Caribbean community-of-practice network capable of addressing environmental and public health problems

The AML has created a unique and effective conduit where the academic community can meaningfully link with the research needs of Caribbean regional institutions, and help create, foster, and support a cadre of engaged professionals interested in researching the link between human and environmental health issues. Most critically, the CEHP program has made a tangible contribution to ameliorating the chronic shortage of qualified professionals to run local and regional laboratories that have to oversee the implementation of public health and environmental programs in this part of the world.

**Figure 2:**
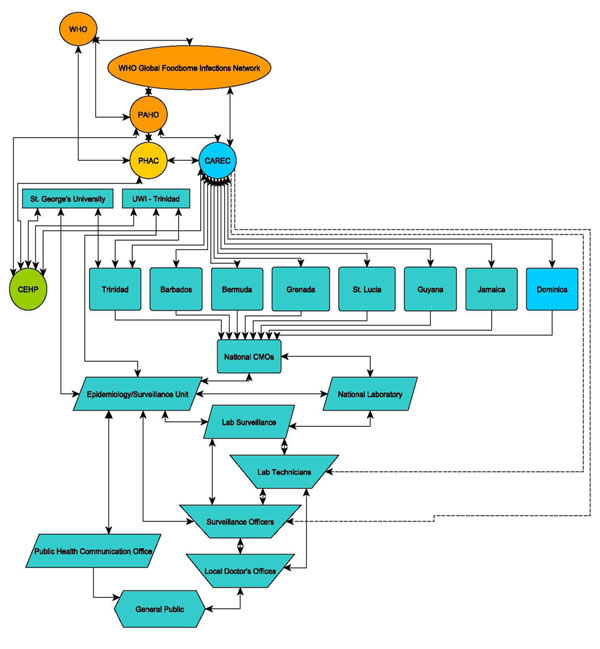
Overview of stakeholders involved in the Burden Of Illness research program.

A most exciting outcome of the CEHP’s efforts to foster and nurture regional and local multi-disciplinary teams is that this approach has generated tremendous interest throughout the region to be involved in each of our core research projects. For example, the POPs study had to be expanded from the originally planned four islands to include an additional twelve countries. The gastrointestinal foodborne BOI study generated much interest and demand such that it had to be expanded from the original target of four countries to eight – and perhaps eventually to ten. The range of stakeholders involved in the BOI study is outlined in **Figure**2.

Due to multiple requests from regional and local governments and their technical agencies, the RWH study research program had to be expanded to include a focus on not only rainwater cisterns, but also seawater and freshwater sources. After consultation with regional academics and a review of regional job market needs, the CEHP’s original goal of developing a single online certificate course in food safety at the University of the West Indies (UWI), St. Augustine campus, had to be expanded to a full MSc degree and diploma evening program that is already oversubscribed and, as a result, has caused UWI to presently limit new students to those only from Trinidad.

In most of the above listed cases, the AML has been a key factor in helping the CEHP to successfully achieve its mandate of building a collaborative network and community of environment and health researchers and graduate students in the Caribbean region. This network, in turn, has served to be the catalyst of several new research studies. For example, this was realized in Grenada with the study on Marine Recreational Water Quality for the Southwestern Coastline conducted by the Windward Islands Research and Education Foundation (WINDREF) in collaboration with an MSPH student of SGU and the Fisheries Division of Grenada. In Dominica, river pollution profiling was conducted jointly by the Ministry of Agriculture and RUSM medical students outlining spatial changes in the level of water quality monitoring (WQM) indicators from the high reaches to the point of entry to the sea. In Barbados, studies were conducted jointly with post-grad students of UWI, (Cave Hill Campus, Barbados), Department of Biological and Chemical Sciences on new and appropriate marine WQM indicators for the tropics as well as resistant strains testing for WQM indicators and specific pathogens.

### Translation of research results

The CEHP has also helped with knowledge translation and technology transfer among public and environmental health professionals in the Caribbean Community (CARICOM). To date, the CEHP program has been able to address significant gaps in the regional knowledge base that are of key importance to regional research users and decision and policy makers. For example, the BOI studies have already begun to guide policies by the Ministries of Health for the testing of pathogens related to foodborne illnesses and have identified ways in which laboratory and community surveillance systems can be enhanced and improved. With the recently developed graduate programs in Agri-Food Safety and Quality Assurance, students will be prepared and equipped to address current and emerging food safety and quality assurance issues that arise in the Caribbean.

Another of the CEHP’s knowledge translation successes can be linked to a study that was initially done in 2004 when the AML was carrying out an attachment in Bermuda. This Bermudian study measured mercury in umbilical cord blood from 42 healthy newborns, and found an arithmetic mean concentration of 41.3 nmol/L (range 5 nmol/L to 160 nmol/L)[19]. This concentration is about 8 times that found in Québec and North America in general [20]. Furthermore, it was estimated that 85% of total mercury measured was in the form of methylmercury (MeHg), which strongly points towards seafood as the main source of mercury exposure during pregnancy. These results were presented to the department of health and a cabinet paper was issued requesting that specific dietary advice be given to the Bermudian population to limit the consumption of large predator fish. In 2010, under the CEHP program, the POPs study was implemented in Bermuda. The arithmetic mean mercury blood concentration in the blood of 49 pregnant women was now found to be 6.6 nmol/L (95% CI of 4.9-8.3). These results seem to provide clear evidence that mercury exposures in the Bermudian population had dropped by a factor of 4 to 5 following the public health advisories issued as a result of the initial finding. These results also illustrate the impact our research efforts can have on key policy makers in the region.

### Development and enhancement of human capabilities

Each CEHP research project has a local human capability development component. For example, for the POPs study, rather than collect samples and carry out analyzes using outside expertise, the CEHP program hired and trained nurses, health professionals, and senior laboratory technicians within each island. These locally hired people were trained in the study’s research methodology and protocols, in research ethics, and in the management of human subjects and bio-samples. They were thus enabled to conduct this research study within their countries on CEHP’s behalf. Similarly, for the BOI studies, local persons, including graduate students from Caribbean universities, were identified and trained to conduct this study in their respective islands. In each island where the BOI study was conducted, all national lab technicians were also trained to identify and report on an expanded list of foodborne pathogens. Further, epidemiologists and practitioners in the field were trained in the collation and statistical analyses of data specific for the BOI study (which involves lab training and two national surveys done during high and low seasons of acute gastrointestinal illness) and other similar research projects.

As a result of the CEHP’s commitment to interdisciplinary research within the context of international collaborations, unique and otherwise unavailable opportunities have been created to leverage students, research, time and resources in support of the program’s objectives. For example, the CEHP program network has allowed graduate students from the partner CEHP universities to receive training and research experience that otherwise would not have been possible. This can be exemplified using the case of the AML technician who was initially chosen from among those who applied to work in the AML in Dominica. Due to the extensive training she received both in Dominica and later Quebec, Canada, she was retained and asked to stay with the lab as it moved to Barbados. There, because of her training and newfound skills, she was able to successful apply to UWI’s MPhil program. She is now working along with one of our core research partners at UWI on a research project to conduct monitoring of chemical contaminants in the marine environment using novel semi-permeable membrane devices (SPMD). Additionally, it was only as a result of the network that was established by CEHP, which brought together researchers from our Northern partners (in this case Trent University in Canada), that she was able to secure funding from the Canada Caribbean Scholarship Programme to pursue a six months training attachment at the environmental toxicological laboratory of Trent University. There she has obtained further hands-on specialized training in the production of SPMDs and the determination of contaminants uptake in fresh and marine waters and analyses for the contaminants. The higher-level skills and assessment sampling methodologies this Dominican technician has obtained to date are now being applied for the first time to research in the Caribbean to help assess chemical pollutants in the marine environment.

In another example, a MSPH student from Laval University was able to have a three (3) month attachment at AML’s ecotoxicological laboratory unit to conduct research on the mercury levels in women working in the fishing industry by hair samples analyses. Of the 46 women sampled, the average measured mercury body-burden was 4.65 *μ*g/g. Among 4 cases, however, exceptionally high hair mercury levels were discovered: 361, 1404, 4012, and 5616 *μ*g/g. Further detailed follow-up investigations of these four women lead to the identification of mercury-containing skin-lightening creams as the source of their high mercury readings. Indeed, one of the creams obtained from one of the cases contained 20,000 ppm of inorganic mercury. For reference, the U.S. FDA stipulates that no more than a trace amount, 1 ppm (0.0001 percent), be found in skin products. All four women received constructive information sessions encouraging them to stop using these skin-lightening creams. In addition, letters explaining the situation were sent out to the Chief Medical Officer (CMO) of Barbados as well as to all of the other CMOs in the CARICOM region. It is hoped that the ministries of health will initiate steps to inform customers about the health risks from using mercury-containing skin-lightening creams and eventually institute bans on these dangerous products. This study also highlighted the need for further studies in exposure pathways and mercury toxicity in humans.

To date, 8 Caribbean and 4 Canadian graduate students have been directly engaged in CEHP related research projects. Specific training opportunities made possible by the presence of the AML in the Caribbean are presented in **Table**2.

**Table 2:** Human resource development and enhancement through the Caribbean Ecohealth Programme’s Atlantis Mobile Laboratory.

Grenada				
**Training**	**# Participants**	**Male**	**Female**	**Comments**

Epidemiology	17	4	13	POPs Research
Ecotoxicology	8	3	5	ELISA Analyses
Research techs.	12	4	8	POPs Research
Lab Analyses	10	4	6	Chem and Biological

**Dominica**				

**Training**	**# Participants**	**Male**	**Female**	**Comments**

Epidemiology	8		8	POPs Research
Ecotoxicology	6	3	3	ELISA Analyses
Research techs.	20	7	13	POPs Research
Lab Analyses	25	11	14	Chem and Biological

**Barbados**				

**Training**	**# Participants**	**Male**	**Female**	**Comments**

Env. Chemistry	22	7	15	Yr 1 Undergraduates
Metals Analyses	4		4	For Research Project
Analytical Chem.	1		1	PhD Research
Mercury Analyses	3		3	Field Research
Micro. Pathogen	3		4	MPhil Research
ViTEK	2		2	MPhil Research

### Development and enhancement of material resource capabilities

A key component of the BOI studies was to strengthen and improve both local laboratory capacity and laboratory technician capabilities to identify additional pathogens. Funding was also made available through the CEHP to the Caribbean Environmental Health Institute (CEHI) located in St. Lucia to strengthen the ability of its laboratory to undertake microbial testing of rainwater harvesting systems. This laboratory is primarily used by CARICOM member states to do environmental and public health monitoring.

## Other outcomes of the CEHP partnership

**Figure 3:**
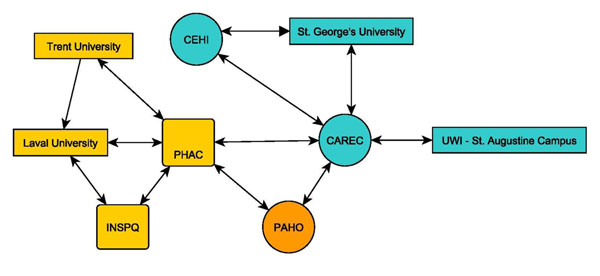
Schematic Diagram of Original Program Partners as of 2007. Legend: Yellow (Canadian), Blue (Caribbean) and Orange (International)

**Figure 4:**
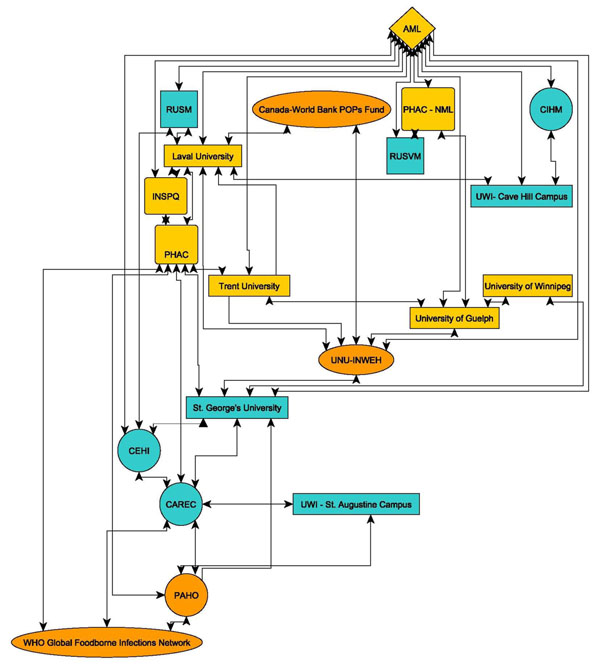
Schematic Drawing of Current CEHP Partners, excluding local groups and national governments, as of 2011.

Over the past four years, the CEHP’s mission to build and develop regional and local networks of public health researchers has resulted in significant increase in the number of core partners. At the initiation of the CEHP program, a core of eight agencies and institutions were involved (see **Figure**3): Laval University, St. George’s University, the University of the West Indies (UWI, St. Augustine Campus), the Public Health Agency of Canada (PHAC), the Institut national de santé publique de Québec (INSPQ), Pan American Health Organization (PAHO), Caribbean Epidemiology Centre (CAREC), and the Caribbean Environmental Health Institute (CEHI). To these initial eight, the CEHP network has grown to include a wide variety of additional organizations, including as a partial list: the Caribbean Institute for Hydrology and Meteorology (CIHM) in Barbados, Ross University School of Medicine (RUSM) in Dominica, Ross University School of Veterinary Medicine (RUSVM) in St. Kitts, the University of Guelph and University of Winnipeg in Canada, and UWI (Cave Hill campus, Barbados) (**Figure**4).

The CEHP program over the past four years has also been able to enter into other partnerships which has resulted in the expansion and enhancement of several of our core research study programs as well as help us fulfil our human resource development commitment to the Caribbean region. For example, an alliance was formed between the United Nations University in Hamilton, Canada, St. George’s University, and Laval University to allow the CEHP to access additional funding for the POPs study from the in Canada-World Bank POPs Fund. The CEHP program would not have been able to obtain the funding without the support and involvement of the UNU. As a result of this partnership, the POPs study was able to expand its research efforts from four islands to cover all 15 CARICOM member states in the Caribbean. Additionally, this collaboration led to the creation of a scholarship opportunity for one of the AML lab technicians to pursue graduate studies in Canada with one of the UNU’s collaborating scientists.

## Challenges and successes

### Challenges

A key challenge the CEHP program has faced is related to delays in obtaining research ethics approval from multiple ethics/institutional review boards (both from Northern and Southern partner institutions) and the timely release of funds from the program funder. The CEHP program has also experienced challenges related to the on-going reorganization of several partner institutions and thus the coming and going of individuals involved in organizing and implementing the various research efforts. Some synergies between the research programs that were initially envisioned were not realized due to the complexity of working with multiple institutions with different timelines, and in some cases training was provided more than once so that the study methodologies would be fresh in the minds of the participating research assistants (for example, if more than one year had passed before the study was able to begin).

Another challenge, yet also a key indicator of the success of this program, has been the expansion of all of the program’s core research topics well beyond its initial mandate (for example, as previously mentioned, 12 instead of 4 countries involved in the POPs study; 8 instead of 4 countries involved in the BOI study; a MSc degree and diploma program at UWI instead of one distance education course; moving the AML to 3 instead of 2 islands).

Given the magnitude of the major regional POPs and BOI studies undertaken in the CEHP and their significant jurisdictional and institutional complexity, many of the studies are just beginning to wrap up in the fourth year of the program. At the other end of the spectrum, not all of the AML-based local demand-driven studies were large enough to warrant publication in peer-reviewed journals. These studies, however, have significant non-trivial value in both informing local decision-makers and encouraging interest in the use of analytical methods to guide and shape policy. Further, given the magnitude of the CEHP’s research program and their local, national, regional and international significance, it has become quite evident that such collaborative research efforts will take much time – indeed much longer than the 5 year timeframe of this grant – to fully realize and see the full impact CEHP program efforts have borne in the Caribbean region. Thus the CEHP program PIs are facing the challenge of thinking how best to leverage its existing human and non-human resources over the remaining period of this grant so as to maximize the benefit of the CEHP program for the people of the Caribbean.

Another key challenge we wish to address is how to integrate the CEHP’s program research findings into place-based polices, particularly national polices. The CEHP research agenda has deliberately focused on filling key knowledge gaps in the CARICOM region. Towards that end, the connections between projects were deemed less critical than the value that would be gained to the region by addressing these missing pieces. Having said that, the CEHP from the outset of the program has seen the benefit of the convergence between the research programs. Early efforts were made, for example, to link the rainwater cistern project to the BOI study in Grenada and Carriacou. In addition, a zoonoses component was added to the POPs study, although originally the former was to have been based out of CAREC’s laboratory in Trinidad. Due to the challenges experienced due to the very different timelines (including difficulties finalizing MOUs and transferring funds), combined with the logistical challenges and timelines inherent in rolling out individual research programs, and not underestimating the institutional challenges caused by the shuffling of key actors in the program, potential synergies have been underdeveloped to date. Nonetheless, over the remaining period of this program, there is the potential to regain some integration by building on the country-level reports created by the research teams. These include detailed POPs and BOI study reports, several of which have already been released. It is also hoped that this integration may be further enhanced through country-level meetings with key policy partners, or via a planned regional forum to be held in the last year of this grant.

The CEHP’s experience provides some insights into the challenges of conducting global health research. The CEHP’s experience illustrates that in order to successfully tackle environmental and public health issues, dynamic teamwork and respectful partnerships are often needed. The CEHP’s wide-ranging network of research team members has facilitated the recruitment and engagement of key government and institutional partners, with each party bringing different resources and capabilities to the group. While it is typically true that the larger institutions and the Northern partners in the CEHP partnership may have access to funds, it is the smaller LMIC partners that are typically more in tune with the critical questions and gaps in research in the region and it is they that tend to have a well-nuanced understanding of who needs to be engaged in order to get ambitious research projects done.

Notwithstanding what has already been said above and what will be highlighted below in the ‘*Successes’* subheading, the CEHP program has found it a challenge to effectively capture all of the successes of the CEHP on paper to our funders and partners. Part of this problem may be that the usually evaluation metrics such as the of number of peer-reviewed journal papers published, number of reports written, and amount of additional co-funded secured are all ill suited to capturing the other less tangible but still highly significant (e.g., the creation of dynamic and sustainable networks of highly motivated professionals within the region, synergies of having multiple agencies and multiple expertise working on current environmental problems which lead to novel and more holistic solutions, etc.) outcomes. With this in mind, the CEHP partners are exploring alternative media wherein it can meaningfully communicate the impact it has truthfully had in the Caribbean region. One option that is currently under development is commissioning of a video-based evaluation of the program and its research efforts. The final product will include an analysis of the overall effort, its successes and failures, and the direct and indirect benefits provided by participation in the collaborative effort. In addition, specific short videos on the BOI, POPs and AML experiences may have particular utility for future knowledge translation efforts. We have no doubt that this program will lead to a significant number of ‘traditional’ indicators of success, such as peer-reviewed journal articles, but are aware that these are not likely to be apparent until 1-2 years after the program ends. Given the regional scope and sensitive nature of the research undertaken, as well as their significant policy implications, it is important that the data are first reported and discussed nationally before they are synthesized into journal articles. Indeed, by working with regional government organizations, such as CAREC, these steps are required.

### Successes

As is true in most LMIC regions of the world, so to in the Caribbean it is important to identify key gaps in scientific knowledge pertaining to the state of environment and health relationships. The dearth of such data in the Caribbean makes it very difficult to engage decision-makers in the quest for policy action that is required to move beyond the status quo. A key advantage of regional studies, such as those that the CEHP program has designed and implemented, is that these studies have been self identified and collaboratively researched by internal and external expertise. Such studies thus have both national and international significance, and participation in these studies is often of particular interest to national governments as it creates a more level playing field in terms of who is impacted by the results and what should be done.

The use of the Atlantis Mobile Lab (AML) in catalyzing new science in the Caribbean region and in providing the equipment and expertise required to conduct innovative studies in a cost-effective way has been a major success of the CEHP program. In addition to carrying out CEHP research projects, the lab has been used by other organizations to carry out other specific analyses. These additional studies were made possible because of the CEHP’s contribution of the lab and its technicians to the studies. The analyses provided to study partners at their request were done on a cost-recovery basis in that the requesting entity was only required to either provide or pay of the cost of replacing materials used to support these research projects. This fact alone was extremely powerful in creating demand for data related to questions of on-going concern. For example, the Soufriere Marine Management Association in St. Lucia was able to pay for sampling of marine and surface water for pathogen and metal testing. Information from that study was presented to the community by the CEHP program manager and the lab technician at a meeting of the SMMA. This has led to increased interest and demand for watershed-based programming to protect surface and marine water quality in the area.

The Environmental Health Department of the Government of Dominica took advantage of the opportunity for low-cost analysis to investigate issues related to marine pollution from land-based activities including inadequate liquid waste disposal practices and metal pollution, as well as seafood safety. In addition, a request from the local government and the indigenous Carib people, led to microbial and chemical analyses being carried out on six (6) rivers, springs and public water supplies in the Carib Territory. After the AML left the island, the government made a formal request for the CEHP to prepare a list of equipment that the government should invest in, in order to create on-island capacity to undertake the ecotoxicological and public health studies that it benefitted from during the AML’s stay. Many of the studies were undertaken in partnership with scientists from the Ross University’s School of Medicine. These localized research partnership were significantly strengthened by the CEHP collaboration.

There has also been a very strong demand for the services of the lab not only from University researchers in the region but also from private consulting firms who are looking to have samples analyzed locally. Currently, most samples must be shipped to the U.S. or Europe for analysis due to the lack of equipment and training professionals within the region.

Through the CEHP human capacity and resource development and enhancement initiatives, it has not only provided the equipment but also has now helped further develop the pool of available highly qualified personnel that can undertake such analysis within the region. The benefits of having a mobile lab in the Caribbean region are clear, perhaps mounted on ship in the future as per the AML’s work in the Arctic, as such a resource not only helps in the promotion of new science, but it can also be used to assist with post-disaster recovery efforts related to ecological change and human health (e.g. water quality testing, active epidemiological surveillance, etc.). Coming out of the intensive interest shown by the region’s governments and the region’s environmental and public health agencies, there is an active interest in expanding the capabilities of the lab to better support environmental and public health monitoring initiatives in the Caribbean.

One area in which the CEHP program has had notable success is in its efforts to tangibly link ecology with human health issues. A good example of this was our one-week Oceans and Human Health (OHH) course held in Barbados in November, 2010. This interdisciplinary field course was open to both mid-career professionals and graduate students and was oversubscribed. Final chosen participants came from throughout the Caribbean region, including Mexico and the U.S. Virgin Islands. This course clearly demonstrated the need to better link the professionals charged with adapting and mitigating threats to our ecosystems with those focused on protecting our health. Course participants included, for example, fisheries scientists, ecotoxicologists, epidemiologists, microbiologists, engineers, environmental scientists and public health professionals.

The CEHP program has lead to the creation of new opportunities in Caribbean region for graduate students and professionals in that in addition to training and skills development, it has also provided fora where these newly highly qualified personnel can be encouraged and meaningful utilized within the region where they are most needed. Multi-year, large-scale research projects, such as the CEHP program, foster interest, excitement and camaraderie for the next generation of global health researchers in both the North and South. The longer they last, the greater the opportunity to build and foster this critical capacity.

## List of abbreviations used

AML: Atlantis Mobile Laboratory BOI – Burden of Illness; CAREC: Caribbean Epidemiology Centre; CARICOM: The Caribbean Community; CEHI: Caribbean Environmental Health Institute (St. Lucia); CEHP: Caribbean EcoHealth Programme; CHUQ: Centre hospitalier universitair de Québec; CMO: Chief Medical Officer; DOWASCO: Dominica Water and Sewage Company; GFN: Global Foodborne Infections Network (WHO); IDRC: International Development Research Centre (Canada); INSPQ: National Institute of Public Health of Quebec; LMIC: Low- and Middle-Income Country; MOH: Ministry of Health; MSPH: Master of Science in Public Health; NGO: Non-Governmental Organization; PAHO: Pan American Health Organization; PHAC: Public Health Agency of Canada; POPs: Persistent Organic Pollutants; RUSM: Ross University School of Medicine (Dominica); RUSVM: Ross University School of Veterinary Medicine (Dominica); SGU: St. George's University (Grenada); TC: Teasdale-Corti; UNU: United Nations University; UWI: University of the West Indies (Trinidad, Barbados); WINDREF: Windward Islands Research and Education Foundation; WQM: water quality monitoring.

## Competing interests

The authors declare that they have no competing interests.

## Authors' contributions

All authors read and approved the final manuscript.
